# Prognostic value of frailty in elderly patients with acute coronary syndrome: a systematic review and meta-analysis

**DOI:** 10.1186/s12877-019-1242-8

**Published:** 2019-08-15

**Authors:** Qingyu Dou, Wen Wang, Hui Wang, Yao Ma, Shan Hai, Xiufang Lin, Ying Liu, Xinjun Zhang, Jinhui Wu, Birong Dong

**Affiliations:** 1 National Clinical Research Center of Geriatrics, Geriatric Medicine Center, West China Hospital, Sichuan University, No. 38, Guoxue Rd, Wuhou District, Chengdu, 610041 China; 2 Chinese Evidence-based Medicine Center and CREAT Group, State Key Laboratory of Biotherapy, West China Hospital, Sichuan University and Collaborative Innovation Centre, Chengdu, Sichuan China

**Keywords:** Frailty, Acute coronary syndromes, Elderly, Prognosis

## Abstract

**Background:**

Frailty is common and associated with poorer outcomes in the elderly, but its prognostic value in acute coronary syndromes (ACS) requires clarification. We thus undertook a systematic review and meta-analysis to evaluate the relationship between frailty and poor prognosis in patients with ACS.

**Methods:**

We systematically searched PubMed, Embase to find literatures which studied the prognostic value of frailty in elderly patients with ACS. Our main endpoints were the all-cause mortality, cardiovascular disease (CVD), major bleeding and readmissions. We pooled studies using random-effect generic inverse variance method, and conducted three pre-specified subgroup analyses.

**Results:**

Of 1216 identified studies, 15 studies were included in our analysis. Compared with the normal group, frailty (HR = 2.65; 95%CI: 1.81–3.89, I^2^ = 60.2%) and pre-frailty (HR = 1.41; 95%CI: 1.19–1.66, I^2^ = 0%) were characterized by a higher risk of mortality after adjustment. Frailty also was associated with increased risk of any-type CVD, major bleeding and hospital readmissions in elderly patients with ACS. The pooled effect sizes in frail patients were 1.54 (95%CI: 1.32–1.79), 1.51 (95%CI: 1.14–1.99) and 1.51 (95%CI: 1.09–2.10).

**Conclusions:**

Frailty provides quantifiable and significant prognostic value for mortality and adverse events in elderly ACS patients, helping doctors to appraise the comprehensive prognosis risk and to applicate appropriate management strategies.

**Electronic supplementary material:**

The online version of this article (10.1186/s12877-019-1242-8) contains supplementary material, which is available to authorized users.

## Background

The accomplishments of cardiovascular prevention have decreased the incidence of acute coronary syndromes (ACS) and have delayed the onset age of ACS. The progresses in the treatment of ACS (dual antithrombotic therapy and invasive strategy, e.g. the drug eluting stent) significantly lowers the mortality of ACS and lead to a swift growth in the portion of elderly patients. With the fast population ageing, the mean onset age of ACS patients rises stably in the last decades [1]. Advanced age is one of the forceful prognosticators of mortality and morbidity of ACS. Age as a crucial prognostic marker is presented in the majority of ACS risk scores, including the Thrombolysis in Myocardial Infarction (TIMI) risk score and the Global Registry of Acute Coronary Events (GRACE) score [2].

Frailty, one of the most important health problems in geriatrics, commonly exists in ACS patients, in part due to mutual risk factors. A frail phenotype representing decreased physiological reserve and increased vulnerability reflects better biological age, therefore, it may cause the heterogeneity in clinical consequences within the elderly patients [3]. Frailty has become a substantial factor in assessment of several special medical situations and been embedded into clinical decision making, such as evaluation of surgical risk and cancer treatment. However, this has not yet become incorporated as part of routine management of ACS.

The well-defined pathways for the management of ACS, largely based on randomized controlled trial (RCT) evidence, may not be generalizable to elderly frail patients. Indeed, to date, there is no international guidelines as to how frail ACS patients should be treated. Previous studies have reported frailty was connected with higher mortality or adverse events in ACS patients with some inconsistent information. We therefore undertook a systematic review and meta-analysis to examine the significance of frailty on ACS prognosis, including mortality, cardiovascular events, major bleeding and readmission.

## Methods

### Data sources

The methods of this systematic review and meta-analysis were performed in accordance with the Meta-analysis Of Observational Studies in Epidemiology (MOOSE) Statement [4] (Additional file 1: Text S1). A comprehensive literature search was performed up to July 1, 2018. The language was restricted to English. The primary sources were the electronic databases of PubMed and Embase, using various combinations of Medical Subject Headings (MeSH) and non-MeSH terms: “Frailty” combined with “Myocardial Infarction”, “Acute Coronary Syndrome” and “Heart Attacks”. Additional file 1: Text S2 presents the full search strategy. All results were exported to EndNote for the removal of duplicates. We also screened reference lists of published reviews to identify additional relevant studies.

### Study selection and data extraction

The titles/abstracts and full texts were screened by two investigators (Qingyu Dou and Wen Wang) independently. Studies met following criteria were included: (1) performed a well-defined cohort design; (2) used frailty as a major exposure in elderly ACS patients; (3) elderly population with the age of 65 or greater (4) displayed hazard ratio (HR) or relative risk (RR) for outcomes with a 95% confidence interval (CI) or provided sufficient information to calculate these data. Exclusion criteria included: (1) case reports, reviews and conference abstracts; (2) insufficient parameters concerning main outcomes. (3) Using only one item (e.g. low gait speed) as a marker of frailty.

The related information and parameters from all included studies were extracted by two investigators including the first author’s name, the year of publication, the place of study, the design of study, population, sample size, follow-up time, assessment methods of frailty, prevalence for frailty/prefrailty, and HR or RR of main outcomes with 95% CI. The main outcomes included all-cause mortality, cardiovascular events (re-infarction and stroke/TIA), composite outcome of death and cardiovascular events, major bleeding and readmission during follow-ups. When a cohort was represented by two or more studies, all available articles were included to make the most complete analyses. Discrepancies were addressed by discussion with a third author (Hui Wang).

### Quality assessment

Two authors independently assessed the quality of studies, and disagreements were re-evaluated by a third author. The Newcastlee-Ottawa Quality Assessment Scale [5] was used to evaluate the quality of the literature. The scale assessed the selection of cohorts, comparability of cohorts and the quality of outcomes by 9 parameters. Scores range from 0 to 9. The quality of studies were graded as follows: good (≥ 8 stars); fair (5–7 stars); and poor (< 5 stars).

### Statistical analysis

We conducted analyses of adjusted and unadjusted estimates separately. We used relative risk (RR) or hazard risk (HR) as the effect measure for the association of frailty and adverse outcomes. HR or RR was pooled using random-effect generic inverse variance method. Statistical heterogeneity among studies were evaluated with chi-squared and I-squared statistics [6]. We explored sources of heterogeneity using three pre-specified subgroup hypotheses: type of patients (frailty vs pre- frailty); type of ACS (ST-segment elevation myocardial infarction vs non-ST-segment elevation myocardial infarction); follow-up time (during hospital/within 1 month vs ≤ 1 year vs > 1 year). Subgroup analyses were performed if there were at least two studies in each subgroup category. We detected publication bias using visually examining symmetry of funnel plots and Egger’s tests [7]. We performed a sensitivity analysis by omitting retrospective studies. All statistical tests will be performed with the STATA14.0 software. All statistical tests are two sided, *P* < 0.05 is considered statistically significant.

## Results

### Search strategy and research characteristics

**Fig. 1:**
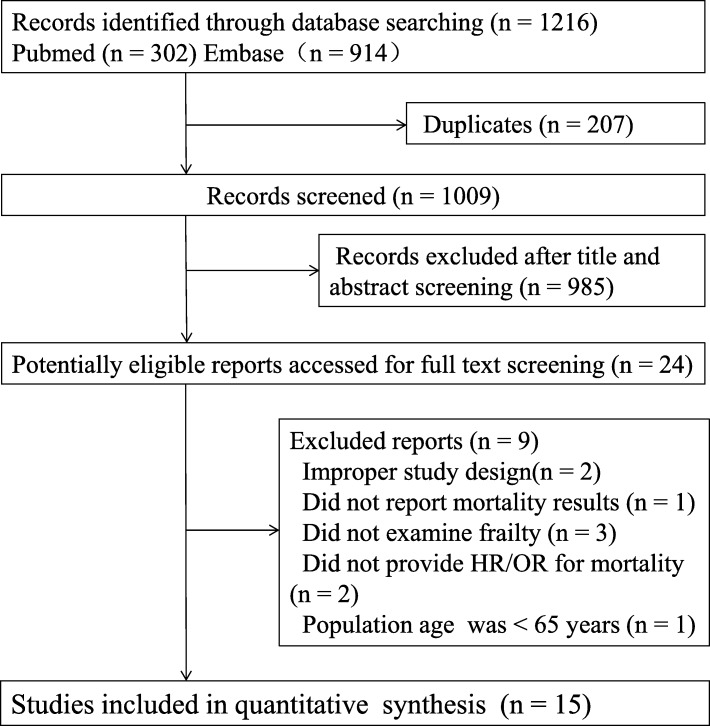
Flow chart of the selection process

Overall, 1216 publications for possible inclusion were revealed by the initial systematic search of the databases. After the removal of duplicates, the remaining titles/abstracts were examined and irrelevant research were excluded, mainly because they were conference abstracts, reviews and case series. Then 24 left articles were carefully reviewed in full texts. Nine of them were excluded for different reasons. Fifteen studies [8–22] including 8554 patients were finally identified. No additional studies were found after manual inspection of the references. Figure 1 showed the flow chart of literature search strategy and detailed reasons of exclusion.

Baseline characteristics of 15 studies [8–22] included are listed in Table 1. The 15 included papers reported data from 13 individual cohort studies. Two papers reported data from one cohort investigating 1 month and 1 year mortality [11, 12]. Another 2 papers studied mortality of average 25 and 56.4 months after ACS from one cohort [17, 18]. There is one retrospective cohort study [19] and the rest are prospective studies [8–18, 20–22]. The studies were conducted in the following countries: Spain, France, Sweden, Canada, Japan, Russia, China and multi-center of the world. All the studies enrolled both men and women with age older than 65 years. The median follow-up time varied from during admission to 56.4 months. Of 15 studies, 1 study enrolled STEMI patients; 4 study enrolled individuals with NSTEMI patients; 6 studies enrolled ACS patients; and 4 studies enrolled type 1 MI.

**Table 1:** Characteristics of included studies on association between frailty and clinical outcomes

Study	Study type	Location	Type of ACS	Frailty measure	Age	Sample Size (n)	No.of Males	Prevalence (frailty,%)	Prevalence (pre-frailty)	Follow-up (mo)
Ekerstad 2011	Prospective	Sweden	NSTEMI	CSHA-CFS	≥ 75	307	257	48.50%	N/A	1
Ekerstad 2014	Prospective	Sweden	NSTEMI	CSHA-CFS	≥ 75	307	257	48.50%	N/A	12
Graham 2013	Prospective	Canada	ACS	EFS	≥ 65	183	123	30.05%	35.50%	12
Sanchis 2014	Prospective	Spain	ACS	Green scores	≥ 65	342	196	48.00%	N/A	Mean 25 (31–72)
Sanchis 2018	Prospective	Spain	ACS	Green scores	≥ 65	342	196	48.00%	N/A	Mean 56.4
Kang 2015	Prospective	China	ACS	CSHA-CFS	≥ 65	352	203	43.20%	N/A	6.3
Sujino 2015	Retrospective	Japan	STEMI	CSHA-CFS	≥ 85	62	36	35.50%	N/A	in-hospital
White 2016	Prospective	Multicentre	UA/NSTEMI	Fried Frailty score	≥ 65	4996	2691	4.70%	23.00%	Mean 17.1 (10.4–24.4)
Alonso 2016 a	Prospective	Spain	type 1 MI	SHARE-FI	≥75	190	115	37.90%	28.40%	1
Alonso 2016 b	Prospective	Spain	type 1 MI	SHARE-FI	≥75	202	121	35.10%	36.60%	in-hospital
Alonso 2017	Prospective	Spain	type 1 MI	SHARE-FI	≥75	234	139	40.20%	28.2%	6
Alonso 2018	Prospective	Spain	type 1 MI	SHARE-FI	≥75	285	171	38.20%	29.80%	12
Kirill 2017	Prospective	Russia	ACS	Computer program of geriatric examination	elderly Senile	633	N/A	35.50%	N/A	12
Blanco 2017	Prospective	France	ACS	EFS	≥ 80	236	N/A	20.8%	28.8%	Mean 15.7
Alegre 2018	Prospective	Spain	NSTEMI	FRAIL scale	≥ 80	532	328	27.30%	38.50%	6

*CSHA-CFS* Canadian Study of Health and Aging Clinical Frailty Scale; *EFS* Edmonton Frail Scale; *STEMI* ST-segment elevation myocardial infarction; NSTEMI, non-ST-segment elevation myocardial infarction; *ACS* acute coronary syndrome; *MI* myocardial infarction; *UA* unstable angina; *CSHA-CFS* Canadian Study of Health and Aging Clinical Frailty Scale; *SHARE-FI* Survey of Health, Ageing and Retirement in Europe Frailty Index; *N/A* not available

The assessment tool of frailty among these studies are as follows: 4 studies [11, 12, 16, 19] used Canadian Study of Health and Aging Clinical Frailty Scale (CSHA-CFS); 4 studies [8, 9, 13, 14] used Survey of Health, Ageing and Retirement in Europe Frailty Index (SHARE-FI) tool; 2 studies [17, 18] from the same cohort used Green Score; 2 studies [10, 15] used Edmonton Frail Scale (EFS); one [20] used Fried Frailty score; and one [21] used FRAIL scale. One last study [22] determined frailty with the help of computer program “optimization of care in geriatrics, depending on the degree of frailty” on the basis of the specialized geriatric examination. All the 15 studies [8–22] reported the prevalence rate of frailty among participants, ranged from 4.7 to 53.2%. Eight studies [8–10, 13–15, 20, 21] reported the rate of pre-frailty ranged from 23.0 to 38.5%.

### Risk of bias assessment

Each study was considered to have adequate methodological quality based on the Newcastle-Ottawa Quality Assessment Scale. These 15 included studies [8–22] were of relatively high methodological quality with their scores ranging from 5 to 9 (mean score = 7.4) (Table 2). Eight studies [9, 10, 12–15, 17, 20] were graded as good quality and the rest 7 studies [8, 11, 16, 18, 19, 21, 22] were graded as fair. The representativeness of the exposed cohort, fully adjusted in the analysis and follow-up time were considered to be the most important indicators for methodological quality.

**Table 2:** Newcastle-Ottawa Score for the included studies

Study	Selection				Comparability		Outcome			Total
Ekerstad 2011	1	1	1	1	1	0	1	0	1	7
Ekerstad 2014	1	1	1	1	1	0	1	1	1	8
Graham 2013	1	1	1	1	1	1	1	0	1	8
Sanchis 2014	1	1	1	1	1	1	1	1	0	8
Sanchis 2018	1	1	1	1	1	0	1	1	0	7
Kang 2015	1	1	1	1	1	0	1	0	1	7
Sujino 2015	0	1	1	1	1	0	1	0	0	5
White 2016	1	1	1	1	1	1	1	1	1	9
Alonso 2016 a	1	1	1	1	1	1	1	0	1	8
Alonso 2016 b	1	1	1	1	1	0	1	0	1	7
Alonso 2017	1	1	1	1	1	1	1	0	1	8
Alonso 2018	1	1	1	1	1	1	1	1	1	9
Kirill 2017	1	1	0	1	0	0	1	1	1	6
Blanco 2017	0	1	1	1	1	1	1	1	1	8
Alegre 2018	0	1	1	1	1	0	1	0	1	6

### Frailty and adverse outcomes in patients with ACS

#### Frailty and mortality in ACS

**Fig. 2:**
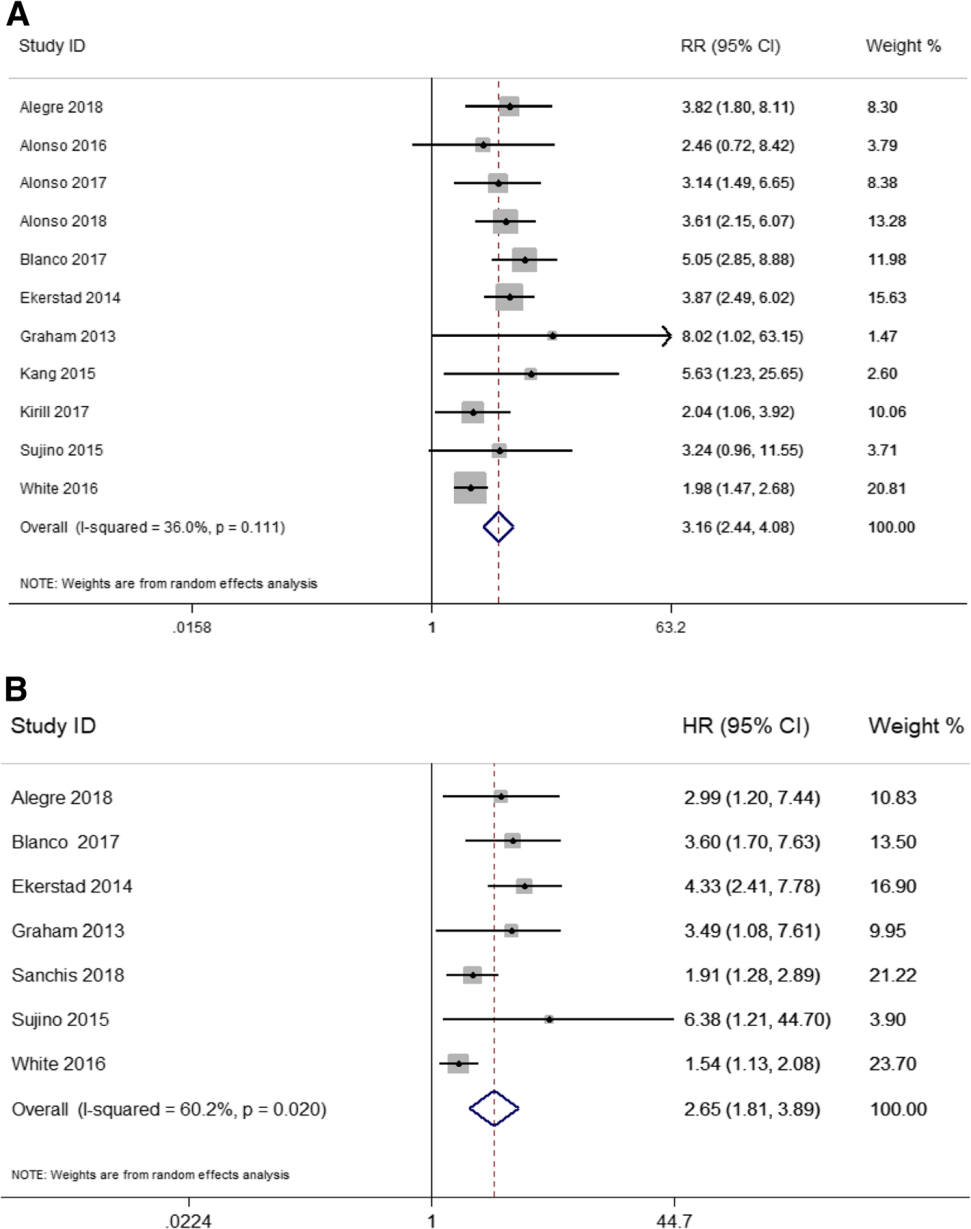
Frailty and mortality in elderly patients with ACS: **a** Unadjusted all-cause mortality during the following-ups; **b** Adjusted all-cause mortality during the following-ups

Using crude data, and taking robust patients as the control, frail group presented a significantly higher risk of mortality (RR = 3.16, 95%CI: 2.44–4.08, I^2^ = 36.0%, *P* = 0.11). After adjusted for potential confounders, compared to robustness, frailty (HR = 2.65; 95%CI: 1.81–3.89, I^2^ = 60.2%, *P* = 0.02) was characterized by a higher risk of mortality. The results were shown in Fig. 2a and Fig. 2b.

#### Frailty and the cardiovascular disease (CVD) risk in ACS

**Fig. 3:**
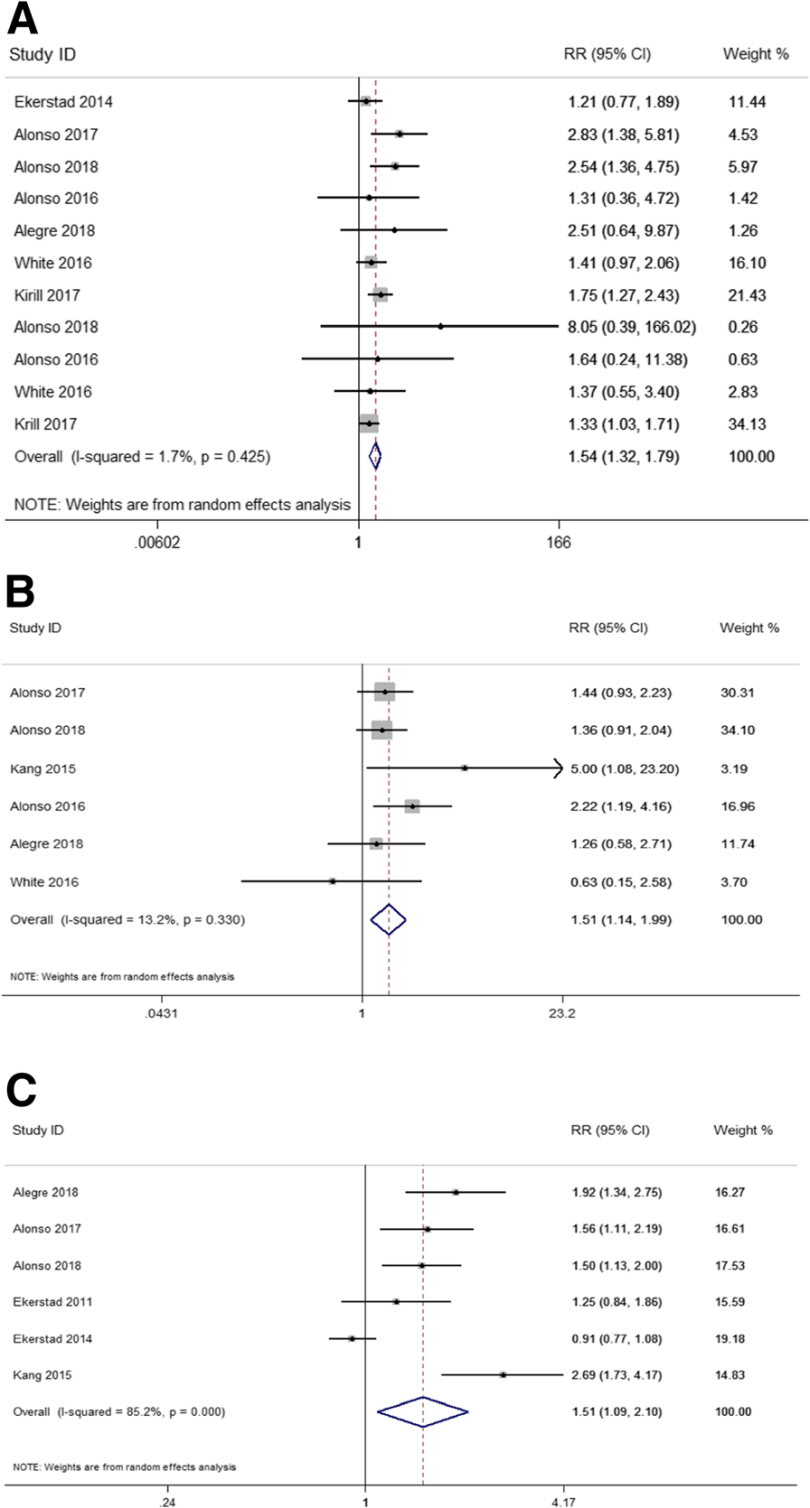
Frailty and any-type cardiovascular disease (CVD), major bleeding and readmission risk in elderly patients with ACS: **a** Unadjusted any-type CVD risk during the following-ups; (**b**) Unadjusted major bleeding risk during the following-ups; **c** Unadjusted readmission risk during the following-ups

Crude data from 7 studies [8, 9, 12, 14, 20–22] were used to examine the influence of frailty on any-type cardiovascular disease (CVD) risk (re-infarction and stroke/TIA) in ACS patients. The pooled RR demonstrated that ACS with frailty resulted in higher risk of any-type CVD risk during the follow-up (RR: 1.54; 95%CI: 1.32–1.79, I^2^ = 1.7%, *P* = 0.425) (Fig. 3a). When analyzing specific CVDs, compared to strong patients, frailty increased the risk of re-infarction of 68% (RR = 1.68, 95%CI: 1.35–2.09, *P* = 0.31, I^2^ = 15.5%) and tended to a 1.6-fold raised risk of stroke/TIA (RR = 1.60, 95%CI: 0.72–3.53, *P* = 0.547, I^2^ = 0%) (Table 3).

**Table 3:** Unadjusted cardiovascular disease (CVD) risk in ACS during following-ups

Outcomes	No. of Studies	Events/Total		RR (95% CI)	*P* value	I^2^
		Frailty	Control			
Any-type CVD						
Reinfarction	7	170/1031	451/4794	1.68 (1.35, 2.09)	0.31	15.5%
Stroke/TIA	3	9/481	61/3906	1.60 (0.72, 3.53)	0.547	0%
Combined mortality with any type CVD						
Combined mortality or reinfarction	2	65/203	30/316	3.39 (2.28, 5.04)	0.846	0%
Combined mortality, reinfarction or stroke/TIA	2	43/180	16/307	4.39 (2.56, 7.51)	0.408	0%

*CVD* cardiovascular disease; *TIA* transient ischemic attack

When CVD was combined with all-cause mortality, 2 studies [9, 14] reported the composite outcome of death and re-infarction, and 2 studies [9, 13] reported the composite outcome of death and re-infarction or stroke/TIA. The corresponding unadjusted pooled RRs were 3.39 (95%CI: 2.28–5.04, *P* = 0.846, I^2^ = 0%) and 4.39 (95%CI: 2.56–7.51, *P* = 0.408, I^2^ = 0%) (Table 3). Due to limited information of included studies, adjusted estimates of other outcomes were not performed.

#### Frailty and major bleeding in ACS

The major bleeding in ACS is defined as patients who had in-hospital intracranial hemorrhage, retroperitoneal bleed, hematocrit drop ≥12%, or need of red blood cells transfusion. Crude data from 6 studies [8, 9, 14, 16, 20, 21] were enrolled in the meta-analysis. We noted that frailty in ACS was associated with the significantly increased risk of major bleeding (RR = 1.51; 95%CI: 1.14–1.99; I^2^ = 13.2%, *P* = 0.33). (Fig. 3b).

#### Frailty and readmission in ACS

Six studies [9, 11, 12, 14, 16, 21] were included in the meta-analysis for the association of frailty in ACS with the risk of readmission. The incidence of readmission was significantly increased by 151% (RR = 1.51; 95%CI: 1.09–2.10, I^2^ = 85.2%, *P* = 0) in frail patients (Fig. 3c).

### Subgroup analyses and publication bias

Sufficient data were available to conduct subgroup analysis only for mortality. Compared with strong patients, pre-frailty significantly increased unadjusted risk of mortality at follow-up (RR = 1.86, 95%CI: 1.28–2.71, I^2^ = 40.1%). Similarly, pre-frailty displayed a significantly increased the risk of mortality after adjustment (HR =1.41; 95%CI: 1.19–1.66, I^2^ = 0%). The incidence of the mortality between frailty and pre-frailty revealed statistical significance before or after adjustment (test for interaction: *P* = 0.022 and *P* = 0.003 respectively). This finding illustrated the group of frailty showed higher mortality than pre-frailty group (Table 4).

**Table 4:** Subgroup analyses of all-cause mortality according to the degree of frailty, follow-up time and type of ACS

Subgroup	No. of Studies	Unadjusted RR (95%CI)	I^2^	P value of interaction	No. of Studies	Adjusted RR (95%CI)	I^2^	P value of interaction
The degree of frailty								
Frailty	11	3.16 (2.44, 4.08)	36.0%	0.022	7	2.65 (1.81, 3.89)	60.2%	0.003
Pre-frailty	4	1.86 (1.28, 2.71)	40.1%		4	1.41 (1.19, 1.66)	0%	
Follow-up time								
During admission/within 1 m	3	3.63 (1.91, 6.90)	0%	0.96	2	3.97 (1.65, 9.57)	0%	
≤ 1 year	7	3.44 (2.67, 4.44)	0%		3	3.80 (2.45, 5.90)	0%	0.18
> 1 year	2	3.06 (1.23, 7.65)	58.8%		3	2.13 (1.32, 3.44)	73.3%	
Type of ACS								
STEMI	2	2.13 (1.11, 4.09)	0%	0.45	2	6.51 (2.01, 21.10)	0%	0.17
NSTEMI	4	2.88 (1.86, 4.47)	58.8%		4	2.63 (1.51, 4.60)	73.5%	

*STEMI* ST-segment elevation myocardial infarction; *NSTEMI* non-ST-segment elevation myocardial infarction; *ACS* acute coronary syndrome

Second, subgroup analyses were conducted according to follow-up time, which can be categorized by three periods, including short-term (during admission or within 30 days), mid-term (follow-up time ≤ 1 year) and long term (follow-up time > 1 year). Overall, frail patients compared with the normal group experienced a similar unadjusted significantly increased risk for short (RR = 3.63; 95%CI: 1.91–6.90, I^2^ = 0.0%), mid (RR = 3.44; 95%CI: 2.67–4.44, I^2^ = 0.0%) and long-term (RR = 3.06; 95%CI: 1.23–7.65, I^2^ = 58.8%) mortality. After adjustment, frail patients still showed significantly increased risk for short (HR = 3.97; 95%CI: 1.65–9.57, I^2^ = 0.0%), mid (HR = 3.8; 95%CI: 2.45–5.90, I^2^ = 0.0%) and long-term (HR = 2.13; 95%CI: 1.32–3.44, I^2^ = 73.3%) mortality. The risks of mortality between subgroups did not show statistical significance (test for interaction: *P* > 0.05). The risk of mortality at different time after ACS were all increased by frailty (Table 4).

We also did the subgroup analyses to learn the connection between the different types of ACS and the mortality. STEMI and NSTEMI frail patients displayed significantly increased risk for mortality without adjustment (RR: 2.13, 95%CI: 1.11–4.09, I^2^ = 0.0% and RR: 2.88, 95%CI: 1.86–4.47, I^2^ = 58.8% respectively). When using the adjusted data, frailty was associated with significantly increased risk of mortality in STEMI (HR = 6.51, 95%CI: 2.01–21.10, I^2^ = 0%) and NSTEMI (HR = 2.63, 95%CI: 1.51–4.60, I^2^ = 73.5%) patients. No statistically significance was detected between subgroups (test for interaction: *P* > 0.05). The results exhibited that frailty was a significant prognostic factor in either STEMI or NSTEMI, regardless of intra-hospital percutaneous coronary intervention (Table 4).

**Fig. 4:**
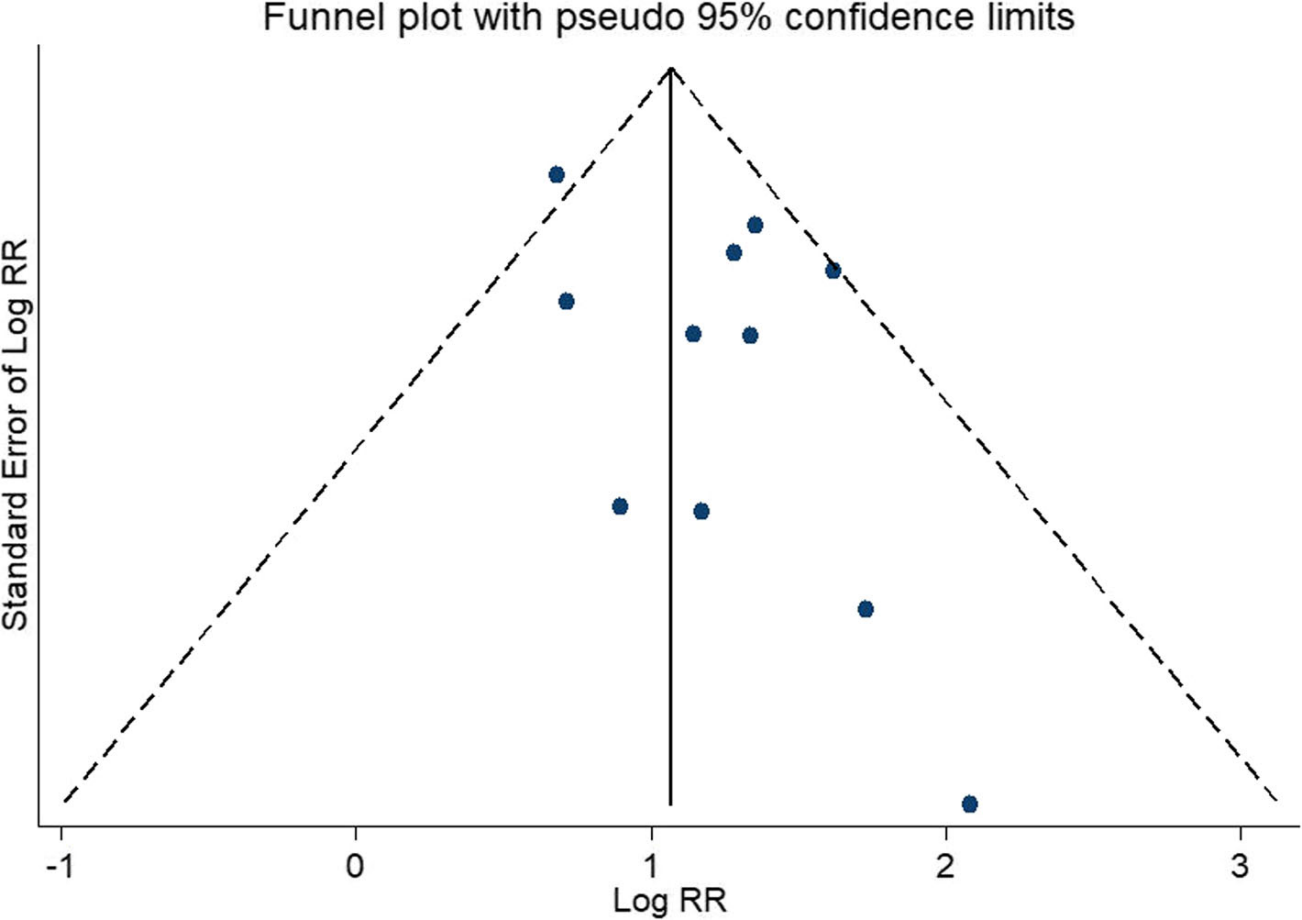
Funnel plots of studies included in the meta-analysis for unadjusted all-cause mortality

No evidence of publication bias was found for the outcomes of unadjusted relative risk of all-cause mortality (Egger’s test *P* = 0.123, Fig. 4). Due to limited numbers of included studies, we did not investigate publication bias for other outcomes.

Sensitivity analysis by omitting Sujino’s retrospective study did not show important changes in the pooled unadjusted (RR =3.17, 95% CI 2.42–4.17, I^2^ = 42.3%) and adjusted estimates (HR =2.65, 95% CI 1.77–3.98, I^2^ = 68.4%).

## Discussion

In the present systematic review and meta-analysis, case series, conference abstracts or reviews, and studies used different exposure or outcome assessment were excluded. All the 15 included studies were of relatively high methodological quality. In the sensitivity analysis, the results were stable after the retrospective study by Sujino was removed. Our results demonstrated in elderly ACS patients, frailty significantly increased the all-cause mortality risk by 2.65-fold, any-type CVD risk by 1.54-fold, major bleeding risk by 1.51-fold and hospital readmissions risk by 1.51-fold.

A previous systematic review reported that the overall prevalence of frailty, in community-dwelling adults aged 65 and older, is on average 10.7%. Moreover, prevalence of frailty increases with age, reaching 15.7% in individuals aged 80 to 84 and 26.1% in those aged 85 or more [23]. The proportion of frailty and pre-frailty in our meta-analysis were significantly higher than community population. Both ACS and frail patients had higher rates of common existed CVD risk factors, like hypertension, type 2 diabetes and lack of exercise. However, regardless of these possible mixed CVD risk factors, frailty itself brings about increased risk of CVD [24]. The pathophysiologic mechanism of frailty, including elevated inflammatory state (interleukin-6 and C-reactive protein [25]), higher markers of thrombosis (D-dimer [25]) and endocrine unbalances (lower insulin-like growth factors − 1 [26]) could act a part in the onset and outcome of ACS. Furthermore, frailty has accelerated biological aging modifications(e.g. higher oxidative stress levels [27], impaired autophagy [28] and shorter telomere length [29] that further give impetus to the development and poor prognosis of ACS.

Frailty could impose obvious influence on the management of elderly ACS patients, especially on revascularization. Since the risk of operational complication rises in frail patients, a less invasive strategy may be preferred. Observational study shows primary percutaneous coronary intervention (PCI) was performed less frequently in patients with frailty compared with their non-frail counterparts [30]. For STEMI patients, timely reperfusion in all patients with ischemia symptoms and persistent ST-segment elevation within 12 h, is the cornerstone of treatment. Studies have demonstrated performing PCI reduced in-hospital mortality, even in patients ≥80 years with frailty [19, 22]. Since our meta-analysis found frailty increased the risk of major bleeding, primary PCI is safer than fibrinolytic therapy especially in high risk elderly STEMI patients with frailty. However, frailty greatly increases all-cause mortality by 6.51-fold in STEMI patients in our study, which may reduce the ability to benefit from interventions. In these extremely frail STEMI patients with a high mortality despite intervention, the death risk should be fully informed and requires a shared decision by doctors and families. The decision not to accept interventional therapy is understandable and reasonable.

The role of an invasive strategy in frail NSTEMI patients is still worth exploring. An early invasive strategy was found to have more benefit in the elderly than younger patients, unless there were extensive and complicated comorbidities [31, 32]. Nowadays, there are studies aimed to judge whether the prognostic impact of PCI in NSTEMI differs across frailty status. The LONGEVO-SCA registry included unselected NSTEMI patients aged ≥80 years. The incidence of cardiac events was more common in patients managed conservatively after adjusting for confounding factors. However, this association was not significant in patients with established frailty criteria [33]. In recent Nuñez’s study [34], a prospective observational study of 270 elderly patients hospitalized for NSTEMI, at a median follow-up of 4.4 years, in patients with Fried ≥3, PCI was associated with a significant reduction of risk of all-cause and cardiovascular-rehospitalizations without reducing all-cause mortality. In 2015 ESC guidelines for the management of NSTEMI, revascularization in elderly patients should be took into consideration after cautious weighing up benefits and risks, including comorbidities, frail state, predicted life expectancy, quality of life, and patient preferences [35].

With regard to medical treatment, as bleeding risk increases with age, comorbidities, polypharmacy and declined renal function, elderly patients are at particular risk of bleeding. In addition to routine evaluation of bleeding risk (HAS-BLED bleeding score), based on our study, we also recommend that the frailty assessment be considered to tailor the individualized antithrombotic treatment for elderly patients. It is essential to reduce bleeding risk according to the degree of frailty; these include employing proper dosage of antithrombotic drugs, avoidance of a glycoprotein (GP) IIb/IIIa-inhibitor; adding a proton pump inhibitor; avoiding the use of non-steroid anti-inflammatory drugs and using radial access whenever possible [36].

Our study found frailty increases both the risk of major bleeding and the risk of cardiovascular events. It is impossible to reduce the incidence of cardiovascular events by increasing the intensity of antiplatelet therapy. Frail patients are also less likely to take angiotensin-converting enzyme inhibitors/angiotensin receptor blockers or β-blockers, since they are more likely to have adverse drug reactions from medical therapy. The frailty as a therapeutic goal intervened by non-pharmacological means is the future hotspot of research. The earlier stage of frailty is reversible and could be remedied. The non-pharmacological interventions (e.g. cardiac rehabilitation, physical exercise, and removing unnecessary medications may postpone and reduce the risk of CVD [37, 38]. A recent study showed patients with diabetes mellitus who have undergone PCI, cardiac rehabilitation participation was associated with significantly reduced all-cause mortality and composite end point of mortality, myocardial infarction, or revascularization [39]. Meanwhile, nutritional supplement with 25–30 g of high-quality protein per meal have slowed or prevented sarcopenia, a manifestation of pre-frailty [40]. In particular, physical activity interventions might play a pivotal role in the prevention of both CVD and frailty. More studies are required to confirm its function and establish standard exercise prescriptions.

### Limitation

There are following limitations in our meta-analysis. First, even though we performed subgroup analysis according to type of ACS and follow-up time, there were not sufficient data to detect the statistical difference between subgroups. Second, the included criterion of our review was based on the age of 65 years or more. However, two studies just recruited participants older than 80 years and another study only included STEMI patients older than 85 years, which may result in the heterogeneity of our studies. Last, none of included studies in our meta-analysis reported results concerning quality of life. Future studies should use more patient-centered consequences such as activity of daily living (ADL) as primary outcome.

## Conclusion

Our study suggests that in elderly ACS patients, frailty assessment should be integrated into the current existing management to better appraise the comprehensive prognosis risk. The identification of frailty help doctors to applicate appropriate management strategies including invasive therapy and antithrombotic medication, and help patients make properly informed choices. Further, the value of frailty as a therapeutic target should be given full attention. Currently, there is little evidence that frailty management could improve outcomes of elderly ACS patients. It is necessary to conduct more studies related the effect of the frailty intervention on elderly ACS prognosis in the future.

## Additional file

Additional file 1: **Text S1**: MOOSE Checklist. **Text S2**: Search Strategy. (DOC 63 kb)

## Data Availability

All data generated or analyzed during this study are included in this published article and its supplementary information files.

## Abbreviations

ACS: acute coronary syndromes
ADL: activity of daily living
CI: confidence interval
CSHA-CFS: Canadian Study of Health and Aging Clinical Frailty Scale
CVD: cardiovascular disease
EFS: Edmonton Frail Scale
GP: glycoprotein
GRACE: Global Registry of Acute Coronary Events
HR: hazard ratio
MeSH: Medical Subject Headings
MI: myocardial infarction
MOOSE: Meta-analysis Of Observational Studies in Epidemiology
N/A: not available
NSTEMI: non-ST-segment elevation myocardial infarction
PCI: percutaneous coronary intervention
RCT: randomized controlled trial
RR: relative risk
SHARE-FI: Survey of Health, Ageing and Retirement in Europe Frailty Index
STEMI: ST-segment elevation myocardial infarction
TIA: transient ischemic attack
TIMI: Thrombolysis in Myocardial Infarction
UA: unstable angina
