# Supplementary material for: Prognostic value of frailty in elderly patients with acute coronary syndrome: a systematic review and meta-analysis
Source: BMC Geriatr. 2019 Aug 15;19:222. doi: 10.1186/s12877-019-1242-8 (PMC6694517; doi:10.1186/s12877-019-1242-8)
Supplement: Supplementary file 1 — Text S1: MOOSE Checklist. Text S2: Search Strategy. (DOC 63 kb) [file 12877_2019_1242_MOESM1_ESM.doc]

**Supplemental Text 1**

**MOOSE Checklist**

**Prognostic value of frailty in elderly patients with acute coronary syndrome: A systematic review and meta-analysis**

| **Criteria** | | **Brief description of how the criteria were handled in the meta-analysis** |
| --- | --- | --- |
| **Reporting of background should include** | |  |
| √ | Problem definition | The fast population ageing and the delay in the age of presentation of ACS is leading to a rapid increase in the proportion of old or very old patients with ACS. Frailty, one of the key health problems in geriatrics, as a prognostic marker has not become embedded as part of routine clinical care of elderly ACS. The influence of frailty on elderly ACS prognosis needs to be investigated quantitatively. |
| √ | Hypothesis statement | Frailty increases the risk of adverse outcomes of elderly ACS. |
| √ | Description of study outcomes | All-cause mortality, cardiovascular events (re-infarction and stroke/TIA), composite outcome of death and cardiovascular events, major bleeding and readmission during follow-ups. |
| √ | Type of exposure or intervention used | Frailty |
| √ | Type of study designs used | Cohort studies, prospective or retrospective were included. |
| √ | Study population | No restriction on study population. |
| **Reporting of search strategy should include** | |  |
| √ | Qualifications of searchers | The credentials of the two investigators Qingyu Dou and Wen Wang are indicated in the author list. |
| √ | Search strategy, including time period included in the synthesis and keywords | PubMed from 1965 –July 2018  EMBASE from 1974 –July 2018  Search strategy see Supplemental Text 2 |
| √ | Databases and registries searched | PubMed and EMBASE |
| √ | Search software used, name and version, including special features | No search software was used. EndNote was used to merge acquired citations and remove duplications. |
| √ | Use of hand searching | We hand-searched the references of extracted papers for additional studies. No additional studies were found. |
| √ | List of citations located and those excluded, including justifications | Details of the literature search process and exclusion reasons are displayed in the Figure 1. Characteristics of included studies were listed in the Table 1. |
| √ | Method of addressing articles published in languages other than English | The language was restricted to English. |
| √ | Method of handling abstracts and unpublished studies | Conference abstracts or unpublished studies were excluded. |
| √ | Description of any contact with authors | There is no need to contact any author. |
| **Reporting of methods should include** | |  |
| √ | Description of relevance or appropriateness of studies assembled for assessing the hypothesis to be tested | Specific inclusion and exclusion criteria were described in the methods section. |
| √ | Rationale for the selection and coding of data | Data extracted from each of the studies were relevant to the population characteristics, study design, duration of follow-up, assessment tool and prevalence of exposure and main outcomes. |
| √ | Assessment of confounding | We conducted analyses of adjusted and unadjusted estimates separately. |
| √ | Assessment of study quality, including blinding of quality assessors; stratification or regression on possible predictors of study results | Each study had adequate methodological quality assessment based on the Newcastle-Ottawa Quality Assessment Scale (Table 2). |
| √ | Assessment of heterogeneity | Heterogeneity among studies were evaluated with chi-squared and I-squared statistics. |
| √ | Description of statistical methods in sufficient detail to be replicated | Description of methods of meta-analyses, heterogeneity analyses, sensitivity analyses and assessment of publication bias were detailed in the methods. |
| √ | Provision of appropriate tables and graphics | There were 4 figures: 1 flow chart for selection process (Figure 1); Two Figures (Figure 2 and 3) described the association between frailty and main outcomes; Figure 4 demonstrated funnel plots of studies included. Four tables were provided, including characteristics of included studies (Table 1), Newcastle-Ottawa Score for the included studies (Table 2), unadjusted CVD risk (Table 3) and subgroup analyses of mortality (Table 4). |
| **Reporting of results should include** | |  |
| √ | Graph summarizing individual study estimates and overall estimate | Figure 2 and 3 |
| √ | Table giving descriptive information for each study included | Table 1 |
| √ | Results of sensitivity testing | Sensitivity analysis by omitting the retrospective study did not show important changes in the pooled unadjusted and adjusted estimates. |
| √ | Indication of statistical uncertainty of findings | 95% CIs were presented with all summary estimates, I^2^ values and results of sensitivity analyses. |
| **Reporting of discussion should include** | |  |
| √ | Quantitative assessment of bias | In sensitivity analysis, the results were stable after the retrospective study by Sujino was removed. |
| √ | Justification for exclusion | Case series, conference abstracts or reviews, and studies used different exposure or outcome assessment were excluded. |
| √ | Assessment of quality of included studies | These 15 included studies were of relatively high methodological quality. |
| **Reporting of conclusions should include** | |  |
| √ | Consideration of alternative explanations for observed results | The adverse influence of frailty on ACS prognosis were discussed from two aspects: shared established CVD risk factors and frailty itself. |
| √ | Generalization of the conclusions | Our study indicates frailty has a substantial impact on elderly ACS prognosis. We noted the lack of studies in Africa. |
| √ | Guidelines for future research | We recommend future studies on the effect of the intervention of frailty on ACS prognosis. |
| √ | Disclosure of funding source | Funding sources of this article were listed in the declarations. |

**Supplemental Text 2**

Search Strategy

Pubmed <1946 to Present>

1. “Acute Coronary Syndrome”[MH]

2. “acute coronary syndrome*”[tiab]

3. ACS[tiab]

4. “Angina, Unstable”[MH]

5. “unstable angina”[tiab]

6. “Myocardial Infarction”[MH]

7. “myocardial infarction”[tiab]

8. “heart attack”[tiab]

9. 1 or 2 or 3 or 4 or 5 or 6 or 7 or 8

10. “Frail Elderly”[MH]

11. Frailty[MH]

12. frail*[tiab]

13. 10 or 11 or 12

14. 9 AND 13

II. Embase <1974 to present>

1. exp acute coronary syndrome/

2. acute coronary syndrome*.ti,ab.

3. ACS.ti,ab.

4. exp unstable angina pectoris/

5. unstable angina.ti,ab.

6. exp heart infarction/

7. myocardial infarction.ti,ab.

8. heart attack.ti,ab.

9. 1 or 2 or 3 or 4 or 5 or 6 or 7 or 8

10. exp frail elderly/

11. exp frailty/

12. frail*.ti,ab.

13. 10 or 11 or 12

14. 9 AND 13
